# Dementia Family Caregivers’ Involvement and Perceived Challenges in Health Care Interactions: Informing the GUIDE Model

**DOI:** 10.1007/s11606-026-10208-w

**Published:** 2026-02-19

**Authors:** Niying Li, Kevin A. Look, Lin-Na Chou

**Affiliations:** 1 Department of Clinical and Administrative Pharmacy, University of Georgia College of Pharmacy, Athens, GA USA; 2 Social and Administrative Pharmacy Division, University of Wisconsin-Madison School of Pharmacy, Madison, WI USA; 3 Department of Physical Therapy and Athletic Training, University of Utah College of Health, Salt Lake City, UT USA

**Keywords:** Alzheimer’s disease, caregiving, caregivers, dementia, health system

## Abstract

**Background:**

Health care interaction tasks, such as making appointments and coordinating care, are vital but often overlooked caregiver tasks. The Guiding an Improved Dementia Experience (GUIDE) Model began in 2024 and aims to support dementia family caregivers through care coordination.

**Objective:**

This observational study described the characteristics of dementia family caregivers and their perceived challenges in performing health care interaction tasks before the start of the GUIDE Model.

**Design:**

Cross-sectional nationally representative survey.

**Participants:**

Primary family caregivers for those with probable dementia from the National Study of Caregiving (NSOC) Round 13 and the National Health and Aging Trends Study (NHATS). We also conducted subgroup analyses examining caregivers’ difficulty performing health care interaction tasks.

**Main Measures:**

Dementia family caregivers were asked whether they had helped with any of the nine health care interaction tasks. Only caregivers who answered yes to the task questions were asked about their difficulty performing the respective health care interaction tasks. We compared the characteristics of caregivers who performed versus did not perform these tasks, as well as those who reported any difficulty versus no difficulty.

**Key Results:**

Our study included 467 caregivers. Most (92.5%) caregivers performed at least one health care interaction task. The most frequently performed health care interaction tasks were speaking to providers (82.2%), making appointments (79.0%), and sitting in on doctor appointments (68.4%). The tasks caregivers rated as being most difficult were transitioning care after a hospital stay (58.9%), coordinating care (43.0%), and handling insurance matters (39.8%). Over half of caregivers assisting after an overnight hospital stay received post-hospital training (58.2%).

**Conclusions:**

Family caregivers commonly perform health care interaction tasks, and their difficulties, particularly transitioning care after a hospital stay, should be recognized and assessed across care settings and providers in the GUIDE Model.

**Supplementary Information:**

The online version contains supplementary material available at https://doi.org/10.1007/s11606-026-10208-w.

## INTRODUCTION

In the USA, more than 16 million family members and other unpaid informal caregivers provided an estimated 18.5 billion hours of care to persons with Alzheimer’s or other dementias.^1^ Caregiver assistance with activities of daily living (ADLs), such as bathing and eating, and instrumental activities of daily living (IADLs), such as managing finances and preparing meals, has been well-documented.^2^ However, ADLs and IADLs do not capture the full scope of caregiver assistance.^3^ A critical group of caregiving tasks is those related to health care,^2,3^ also referred to as the “invisible work.”^4^ The literature uses varying terminology to describe these health care-related tasks (Supplementary Table S1). Nevertheless, there is consensus that these tasks are related to interacting with the health care system (e.g., making appointments, speaking to providers, coordinating care across different providers), and others are related to medical or nursing care (e.g., keeping track of medications, wound care, giving injections).

Health care interaction tasks are particularly important because navigating and interacting with the fragmented and complex health care system in the USA adds extra burden to caregivers.^5,6^ An Alzheimer’s Association Special Report survey indicated that 70% of dementia family caregivers reported that coordinating care (including scheduling appointments and attending doctor visits) is stressful, and 50% of the respondents reported that navigating care (including care coordination, behavioral symptom management, and access to support services) is stressful.^7^ The complexity of the US health care system that older adults need to deal with has been increasing over the years. From 2000 to 2019, the proportion of Medicare beneficiaries who saw five or more physicians annually in the outpatient setting increased by 72%.^8^ The new Alzheimer’s disease anti-amyloid drug approved by the US Food and Drug Administration, lecanemab, also necessitates significant caregiver interactions with the health care system, such as scheduling and coordinating diagnostic tests for drug eligibility and subsequent biweekly infusions, and adds burden to dementia family caregivers.^9^

There is a relatively small but growing body of literature on the challenges faced by dementia family caregivers in health care interaction tasks. A previous study on dementia caregivers who assisted with health care interaction tasks examined the prevalence of caregiver difficulties (physical, financial, and emotional) and observed greater challenges among dementia caregivers compared to non-dementia caregivers.^10^ Some studies have explored dementia caregivers’ challenges in managing health insurance for care recipients,^11,12^ preparing for and accompanying care recipients in health care visits,^13^ and communicating with health care professionals.^14^ Other studies have examined caregivers’ challenges in helping care recipients access care and use telehealth during COVID-19, when caregivers had to navigate limited services and technological barriers.^15,16^

To address challenges in care coordination faced by dementia family caregivers, the Centers for Medicare & Medicaid Services announced a new care-delivery model, the Guiding an Improved Dementia Experience (GUIDE) Model, which began in July 2024 and will run for 8 years.^17^ Providers participating in the GUIDE Model will deliver supportive services to people with dementia and their unpaid family caregivers, including comprehensive, person-centered assessments and care plans, care coordination, and 24/7 access to a support line.^17^

To our knowledge, no study has specifically examined dementia caregivers’ difficulties with health care interaction tasks using nationally representative data. This descriptive study aims to compare the characteristics of dementia family caregivers who performed health care interaction tasks with those who did not, and to examine differences between caregivers who reported difficulty with these tasks and those who did not. The findings can guide CMS GUIDE Model implementation and provide directions for evaluating its effectiveness.

## METHOD

### Data

This study used data from two surveys, the National Study of Caregiving (NSOC) Round 13 and the linked National Health and Aging Trends Study (NHATS).^18^ The NSOC is a study of family and other unpaid caregivers who helped older adults with physical and cognitive limitations in daily activities. NHATS participants are a nationally representative sample of Medicare beneficiaries aged 65 years and older. NHATS participants were eligible for NSOC if they received assistance in the past month with self-care or mobility activities, or with household activities for health or functioning reasons. We used Round 13 survey data because the survey was conducted between June 2023 and May 2024, before the start of the CMS GUIDE Model in July 2024.

Once eligible NHATS participants were identified, they were asked to nominate up to five caregivers to contact for interviews. Five caregivers were randomly selected for older adults with more than five eligible caregivers. Caregivers were eligible for NSOC if they were family members or unpaid non-relatives who assisted the NHATS participants in the past month with mobility, self-care, household tasks, or transportation; in the past year with money matters (other than bills, banking); or helped with medical activities such as handling health insurance. Upon obtaining oral consent, a telephone interview was conducted with the caregivers. Of 2632 living sample persons in NHATS Round 13, 5456 family caregivers were eligible to be interviewed by NSOC. Non-response could be due to NHATS participants not providing contact information for their caregivers, or to caregivers refusing to be interviewed or not being located. The NSOC Round 13 study interviewed 2906 caregivers of living NHATS participants and 265 caregivers of deceased participants in the Last Month of Life sample. The final response rate was 58.2%.^19^

### Study Population

**Figure 1:**
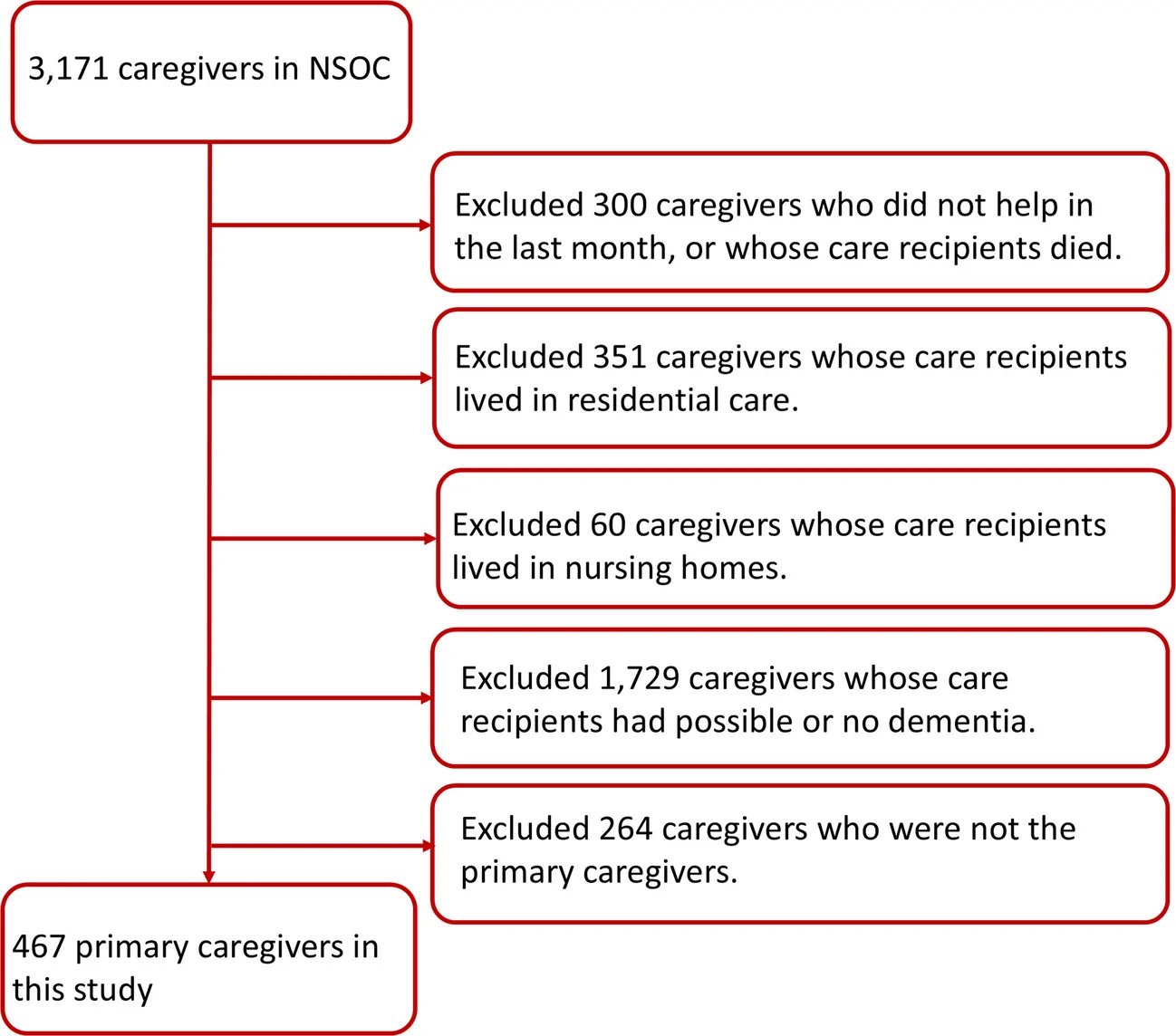
Flowchart of caregiver cohort selection (NSOC Round 13)

We restricted the analytic sample to family caregivers of living, community-dwelling older adults with dementia. Following previous studies,^4,20^ dementia in NHATS and NSOC refers to probable dementia, which NHATS determined by self-report of a dementia diagnosis, a score indicating dementia on the AD8 Dementia Screening Interview, or performance on cognitive tests of memory, orientation, and executive function.^21^ Figure 1 shows the caregiver cohort selection. We first excluded 300 caregivers who did not help in the last month or whose care recipients died between Round 12 and Round 13. We further excluded 351 caregivers helping older adults living in residential care, 60 older adults living in nursing facilities, and 1729 caregivers whose care recipients had possible or no dementia. In situations where care recipients had multiple caregivers, we identified the primary caregiver as the one with the longest caregiving hours in the last month, following the approach of previous research.^22,23^ For caregivers who provided help but did not report caregiving hours, hours were imputed using the NHATS technical document.^24^ If there is a tie in caregiving hours, we followed the approach by Hu and colleagues, using the number of ADL and IADL care activities to break the tie.^25^

### Variables

#### Health Care Interaction Task

Dementia family caregivers were asked in the past year whether they had helped with any of the nine health care interaction tasks: assistance with (1) speaking to providers, (2) making appointments, (3) logging into an online account to see the care recipient’s medical information or test results, (4) coordinating the care recipient’s care across more than one provider, (5) changing or adding a health insurance or prescription drug plan, (6) handling other insurance matters (e.g., looking for what is covered, comparing plans), (7) helping after an overnight stay at a hospital, (8) getting online for telehealth visits with the regular doctor, and (9) sitting in on doctor appointments. Answers to these questions were in yes/no format. A detailed description of the items is in Table 1. We classified family caregivers into two categories (No—answered no to all nine items, Yes—answered yes to any task question).

**Table 1:** Description of Items Related to Health Care Tasks and Difficulties in Performing Health Care Interaction Tasks in NHATS and NSOC Round 13

Items related to health care tasks	Items related to difficulties in performing health care tasks
In the last year, did you ever speak to or e-mail any of {SP}’s medical providers about {his/her} care?	Was it very, somewhat, a little, or not at all difficult for you: coordinating {his/her} health care?
In the last year, did you ever make appointments for {SP} with any medical provider?	
In the last year, did you log into an online account to see {SP}’s medical information or test results?	
In the last year, did you coordinate {SP}’s care across more than one provider? This includes talking to different care providers to make sure they agree about {his/her} treatment plan	
In the last year, did you help {SP} change or add a health insurance or prescription drug plan? For example, changed or helped {him/her} change a Medicare supplemental plan or prescription drug plan, or helped {him/her} decide to join or leave a managed care plan	Was it very, somewhat, a little, or not at all difficult for you: handling {his/her} health insurance matters?
In the last year, did you handle any other health insurance matters related to {SP}’s medical care? This includes contacting Medicare or another insurer to find out what is covered, comparing plans or providers, finding out about bills, or filing a claim	
In the last year, did you help {SP} after an overnight stay at a hospital?	Was it very, somewhat, a little, or not at all difficult for you: transitioning {his/her} care after a hospital stay?
In the last year, did the {OP} most often sit in on {SP}'s doctor visits?	No difficulty question asked
In the last year, did the {OP} who most often sat in on {SP}'s doctor visits ever help {you/SP} with getting online for a telehealth visit?	No difficulty question asked

*NSOC*, National Study of Caregiving; *NHATS*, National Health and Aging Trends Study; *SP*, sample person, referring to the care recipient; *OP*, other person, referring to the caregiver

#### Difficulty in Performing Health Care Interaction Task

Only caregivers who answered yes to the task questions were asked about their difficulty level performing the respective health care interaction tasks. Three types of difficulty were assessed in the survey: coordinating health care, handling health insurance matters, and transitioning care after a hospital stay (Table 1). Response options included very difficult, somewhat difficult, a little difficult, and not at all difficult. For analysis, responses were dichotomized as “no difficulty” versus “any difficulty” (i.e., a little, somewhat, or very difficult).

#### Caregivers Characteristics

Other variables of interest are selected based on previous literature on caregivers’ challenges in performing health care-related tasks,^11,12,26^ including caregiver age (17–59, 60–69, 70 and older), sex (female, male), race and ethnicity (Non-Hispanic White, Black, Hispanic and Others), relationship to care recipient (kin caregivers, including spouse, partner, child, stepchild, and other relatives; and non-kin caregivers, including friend, neighbor, and other non-relatives), education (less than or equal to high school, some college and above), whether the caregiver lived with the care recipient, caregivers’ hours spent in caregiving in the last month, whether the caregiver had spent more than 4 years in caregiving, the number of care recipients’ self-reported chronic conditions (including heart attack, heart disease, high blood pressure, arthritis, osteoporosis, diabetes, lung disease, stroke, and cancer), the number of caregivers’ self-reported chronic conditions (including heart attack, heart disease, high blood pressure, arthritis, oeteoporosis, diabetes, lung disease, cancer,  serious difficulty seeing and serious difficulty hearing), whether the care recipient used telehealth with regular doctor, and whether the care recipient was covered under the Medicare Prescription Drug Program (or Medicare Part D, in which Medicare enrollees pay for outpatient prescription medications through private insurance plans approved by Medicare), Medigap (or Medicare Supplement Insurance, which is private insurance that helps cover costs not paid by Original Medicare), or Medicaid (a government program that provides health coverage to people with limited income and resources). Only caregivers who assisted with overnight hospital stays were asked whether medical providers at the hospital provided them with training to manage care recipients’ post-hospital care. We collapsed subcategories of caregiver age, race and ethnicity, education, and relationship to care recipient per the NHATS requirement that single unweighted cell counts smaller than five need to be collapsed.^27^

### Analysis

We first presented and compared the characteristics of caregivers who performed health care interaction tasks with those who did not. Among the subgroups who performed the tasks, for each difficulty item, we further presented and compared the characteristics of caregivers between those with and without difficulty. Statistical differences between groups were evaluated using Rao-Scott chi-square tests for categorical variables and adjusted Wald tests for continuous variables, respectively.

All statistical analyses were conducted using STATA 18 (StataCorp, College Station, TX). The complex survey design and weights of NSOC Round 13 were used in all analyses with the STATA *svy* commands. We used STATA’s *singleunit (centered)* option to account for strata with a single primary sampling unit (PSU), which centers the PSU-level variance contributions at the grand mean. Statistical significance was set a priori at two-tailed *p* < 0.05. This study used publicly available deidentified data, so this study was exempt from Institutional Review Board review.

## RESULTS

The study sample included 467 dementia family caregivers (weighted: 3,146,025). The characteristics of the caregivers are presented in Table 2. Most caregivers (92.5%) performed at least one health care interaction task, with an average of 4.5 tasks. The caregivers were mainly females (66.1%) and lived with the care recipients (67.7%). We found no statistically significant group differences in caregiver demographics, caregiver chronic conditions, or care recipient health insurance coverage. Caregivers who performed any health care interaction tasks were more likely to be kin caregivers (80.3% vs. 36.8%, *p* = 0.002), to have provided care over 4 years (77.4% vs. 44.0%, *p* = 0.018), and to have spent over three times as many caregiving hours in the last month (216.0 vs. 70.5 h, *p* < 0.001). Caregivers who performed any health care interaction tasks were more likely to care for recipients with a greater number of chronic conditions (3.1 vs. 2.2, *p* = 0.034). Another notable finding was that most doctor visits were conducted exclusively in person (81.6%), whereas only 12.3% involved telehealth (online only or hybrid), and 6.1% of care recipients did not have a regular doctor.

**Table 2:** Weighted Characteristics of Dementia Family Caregivers Based on Health Care Interaction Tasks (NSOC Round 13)

	No health care interaction task Weighted: 236,743 Weighted %	Any health care interaction task Weighted: 2,909,281 Weighted %	Total Weighted *N*: 3,146,025 Weighted %	*p*-value
Overall	7.5%	92.5%	100.0%	
Caregiver age				
17–59	51.8%	39.9%	40.8%	0.57
60–69	20.3%	33.8%	32.7%	
≥ 70	27.9%	26.3%	26.4%	
Caregiver sex				
Male	38.9%	33.5%	33.9%	0.70
Female	61.1%	66.5%	66.1%	
Caregiver race and ethnicity				
Non-Hispanic White	66.6%	54.5%	55.4%	0.41
Black	6.1%	15.5%	14.8%	
Hispanic and Others	27.3%	30.0%	29.8%	
Co-residence with care recipient				
No	55.2%	30.4%	32.3%	0.069
Yes	44.8%	69.6%	67.7%	
Relationship to care recipient				
Kin caregiver	36.8%	80.3%	77.0%	0.002
Non-kin caregiver	63.2%	19.7%	23.0%	
≥ 4 years of caregiving				
No	56.0%	22.6%	25.1%	0.018
Yes	44.0%	77.4%	74.9%	
Caregiver education				
≤ high school	36.1%	36.8%	36.7%	0.96
≥ some college	63.9%	63.2%	63.3%	
Care recipient under Medicare Part D				
No	26.4%	34.7%	34.0%	0.58
Yes	73.6%	65.3%	66.0%	
Care recipient under Medigap				
No	45.7%	43.3%	43.5%	0.88
Yes	54.3%	56.7%	56.5%	
Care recipient under Medicaid				
No	61.2%	62.5%	62.4%	0.94
Yes	38.8%	37.5%	37.6%	
Telehealth use with regular doctor				
No regular doctor	--*	5.6%	6.1%	0.63
In person only	76.8%	82.0%	81.6%	
Online only or hybrid	--*	12.3%	12.3%	
Number of total health care interaction tasks (mean, SE)^†^	--	4.5 (0.2)	--	--
Caregiving hours last month (mean, SE)	70.5 (70.0)	216.0 (243.2)	205.0 (237.8)	< 0.001
Care recipient’s number of chronic conditions (mean, SE)^‡^	2.2 (1.4)	3.1 (1.7)	3.1 (1.7)	0.034
Caregiver’s number of chronic conditions (mean, SE)^§^	2.0 (1.9)	1.8 (1.6)	1.8 (1.6)	0.71

^*^Estimates based on too few cases (< 5 unweighted cell count) may not be reported, per NHATS

^†^*SE*, standard error

^‡^Chronic conditions were self-reported, including heart attack, heart disease, high blood pressure, arthritis, osteoporosis, diabetes, lung disease, stroke, and cancer

^§^Chronic conditions were self-reported, including heart attack, heart disease, high blood pressure, arthritis, osteoporosis, diabetes, lung disease, cancer, serious difficulty seeing, and serious difficulty hearing

**Figure 2:**
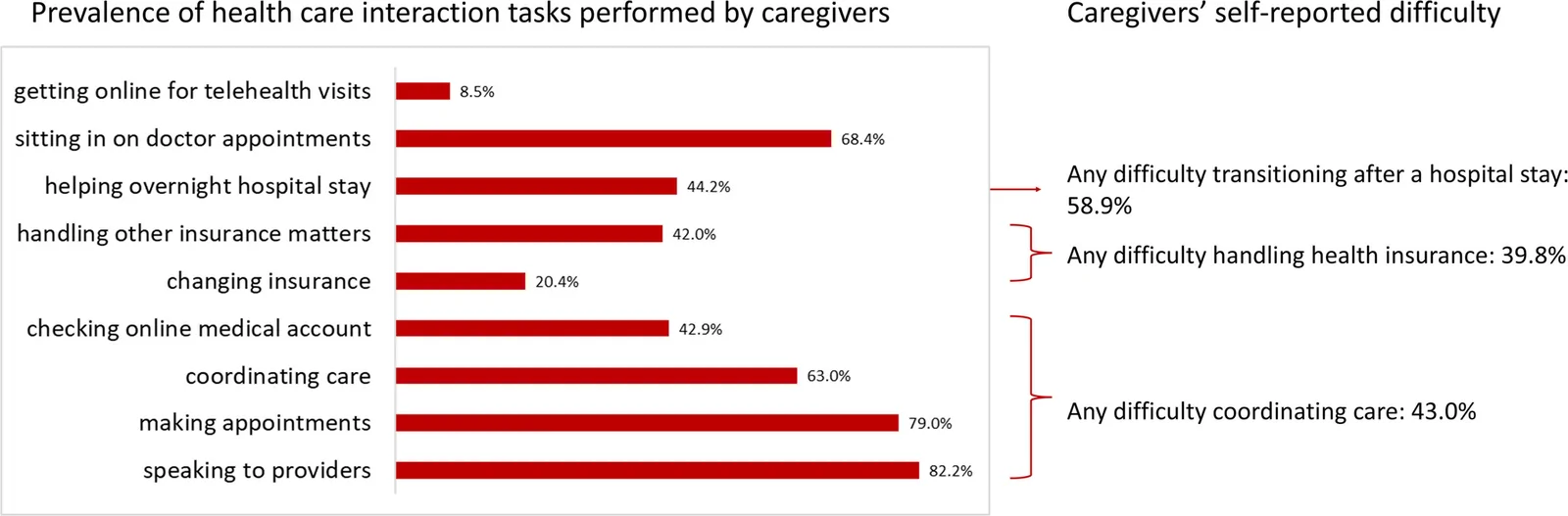
Weighted prevalence of dementia family caregivers’ assistance with health care interaction tasks and caregivers’ self-reported difficulty in performing these tasks (NSOC Round 13)

Figure 2 shows the percentage distribution of dementia family caregivers’ assistance with health care interaction tasks and their corresponding difficulty. The most frequently performed health care interaction tasks were speaking to providers (82.2%), making appointments (79.0%), sitting in on doctor appointments (68.4%), and coordinating care (63.0%). The tasks caregivers rated as most difficult were transitioning care after a hospital stay (58.9%), coordinating care (43.0%), and handling insurance matters (39.8%). Caregiver assistance with getting online for telehealth visits was low (8.5%).

Supplementary Tables S2a–S2c present subgroup analyses of the characteristics of dementia family caregivers who reported no difficulty versus those who reported any difficulty for each difficulty item. We did not observe statistically significant differences in the subgroup analyses, except that caregiver race and ethnicity were significantly associated with self-reported difficulty coordinating care (Table S2a, *p* = 0.028). Hispanic and other caregivers were more likely to report difficulty coordinating care, whereas non-Hispanic White and Black caregivers were less likely to do so, and caregiver age was significantly associated with self-reported difficulty handling health insurance (Table S2b, *p* = 0.040). Younger caregivers aged 17–59 were more likely to report difficulty (59.8%) than those aged 60–69 (21.9%) and 70 or older (18.3%). Over half of caregivers who assisted with overnight hospital stays received training in post-hospital care from health care providers (Table S2c, 58.2%). However, receiving training was not significantly associated with self-reported difficulty transitioning after an overnight hospital stay (55.0% vs. 62.8%, *p* = 0.41).

## DISCUSSION

Using nationally representative data, we examined the challenges dementia family caregivers face when interacting with the health care system. Our study showed that caregivers faced difficulties mainly transitioning after a hospital stay, handling health insurance matters, and coordinating care. Caregivers who performed any health care interaction tasks spent more than three times as many hours on caregiving in the last month as those who did not, suggesting that these often-overlooked tasks are complex and time-consuming.

We did not observe statistically significant differences in caregiver age, sex, education, race and ethnicity, number of chronic conditions, or the care recipient’s health insurance type between those who performed any health care interaction tasks and those who did not. This suggests that these tasks may be undertaken by caregivers regardless of their demographics. We also found that caregivers’ likelihood of performing health care interaction tasks does not appear to depend on their health conditions or care recipients’ health insurance, but depending on the care recipients’ health conditions. This indicates that care recipients’ health conditions drive health care interaction tasks.

We observed generally low telehealth use among care recipients and caregiver assistance with telehealth. Although telehealth expanded rapidly during COVID-19, a nationally representative survey showed that respondents were willing to continue using telehealth after COVID-19 but preferred in-person care.^28^ It is unclear from publicly available documentation whether the GUIDE Model explicitly incorporates telehealth.^17^ Future research should examine whether and how participating programs implement caregiver support via telehealth within the Model.

Our study showed that 58.2% of dementia family caregivers received training from providers after helping with overnight hospital care. This is lower than a previous study using the 2017 NSOC data examining all kinds of family caregivers (59.1%).^29^ Over 40 states in the USA have passed the Caregiver Advise, Record, and Enable (CARE) Act since 2016 to increase hospital support for patients and caregivers during a hospital discharge.^30^ However, our study showed that although over half of caregivers received training, they still expressed difficulties transitioning after a hospital stay. Future studies could investigate what makes post-hospital stay transition difficult from the caregivers’ perspectives and their needs for further assistance.

Previous studies supported our finding that caregivers faced difficulty handling health insurance matters. The Alzheimer’s Association Special Report survey indicated that 32% of dementia family caregivers would like assistance navigating various insurance options.^7^ A recent pilot study on a user-centered decision aid to help Medicare beneficiaries choose their Part D plans found that it helped them make informed choices.^31^ Future research could test the usability of the decision aid for dementia caregivers, as our data showed 42.0% of caregivers helped with handling insurance matters, such as finding out what is covered, comparing plans, and changing insurance plans.

This study has several limitations. First, in the subgroup analyses, there may have been a true difference in characteristics between those who reported difficulty and those who did not; however, our study was underpowered to detect it. Second, health literacy, or the ability to obtain, process, and understand health information and services, is also linked to caregiver difficulty in communicating with providers and navigating the health care system.^32^ However, the NSOC data do not contain variables measuring health literacy, although we used caregivers’ education as a proxy.^33^ Future research and interventions could consider caregiver health literacy to identify and reduce their difficulty performing health care interaction tasks. Third, we collapsed subcategories of caregiver age, race and ethnicity, education, and relationship to care recipient per the NHATS requirement that single unweighted cell counts smaller than five be collapsed. For example, spousal caregivers and adult-child caregivers differ in the type of tasks they assist with.^34^ Collapsing them into a single group masks the differences in this group. Future studies could evaluate whether the GUIDE Model components (training, navigation, respite care) differ for spousal versus adult-child caregivers based on the tasks they perform. Fourth, we did not conduct logistic regression to explore the relationship between caregiver characteristics and task difficulty in subgroup analyses due to the small sample size in each subgroup, as per the ten events per variable rule.^35^ Studies with bigger sample sizes are needed for future research. Fifth, the generalizability of our findings may be limited for individuals residing in nursing homes or residential care facilities. Although caregivers continue to play a central role in health care interactions on behalf of family members in these settings, future research is needed to explore their unique needs and challenges.

## CONCLUSION

This study explored the characteristics of dementia family caregivers and their difficulty in performing health care interaction tasks and set the stage for the GUIDE Model. Caregiver difficulty was found mainly in transitioning care after a hospital stay, handling health insurance matters, and coordinating care. It is critical to recognize and address caregivers’ difficulties navigating the health system across different care settings. Future research should examine how implementing the GUIDE Model influences caregivers’ engagement in health care interaction tasks and whether it reduces the challenges identified in this study.

## Supplementary Information

Below is the link to the electronic supplementary material.

ESM1 (DOCX 33.6 KB)

## Data Availability

Data used in this study are available at https://www.nhats.org/researcher/nhats.
