# Supplementary material for: Dementia Family Caregivers’ Involvement and Perceived Challenges in Health Care Interactions: Informing the GUIDE Model
Source: J Gen Intern Med. 2026 Feb 19;41(13):3771–8. doi: 10.1007/s11606-026-10208-w (PMC13631173; doi:10.1007/s11606-026-10208-w)
Supplement: Supplementary file 1 [file 11606_2026_10208_MOESM1_ESM.docx]

| **Authors** | **Data** | **Definition** |
| --- | --- | --- |
| Polenick at al (2017) | 2011 National Health and Aging Trends Study and National Study of Caregiving | Medical care activities:   (1) Health System interaction tasks:   Ordering medications, scheduling appointments, speaking to providers, helping to change/add insurance or drug plans and other insurance matters.   (2) Medical/nursing tasks:   Giving shots/injections, caring for skin wounds/sores, caring for teeth/dentures, foot care, managing medical tasks (e.g. ostomy care, IVs, testing blood), keeping track of medications, helping with prescribed exercises, assisting with a special diet. |
| Giovannetti et al (2011) | Guided Care randomized trial | Health care tasks:  Obtaining medications, planning medication schedules, administering medication, deciding to change medication, managing medical bills, scheduling medical appointments, arranging transportation, getting information, following the recommended diet, monitoring patients’ health, obtaining medical equipment, and obtaining community services. |
| Wolff et al (2016) | 2011 National Health and Aging Trends Study and National Study of Caregiving | Health care activities:   (1) Assistance with health system logistics:   Making appointments, ordering medications, handling insurance issues   (2) Assistance with health management:   Diet, foot care, skin care, exercise, dental care. |
| Moorman and Macdonald (2013) | 1998-2002 Cash and Counseling Demonstration and Evaluation Study | Nursing tasks:  Helping with ventilator, feeding tube, urinary catheter, colostomy bag, pressure sores, foot care, medications, vital signs, blood sugar, and range of motion. |
| Noureldin and Plake (2017) | 2011 National Health and Aging Trends Study and National Study of Caregiving | Medication management tasks:  Ordering, keeping track of medications, and giving shots/injections. |
| Riffin et al (2017) | 2011 National Health and Aging Trends Study and National Study of Caregiving | Health system logistics:  Making appointments, ordering medications, managing insurance, speaking with medical providers, and keeping track of medications.  Health management tasks:  Diet, foot care, skin care, exercise, and dental care. |

**Supplementary Table S1. Examples of different definitions of health care-related tasks in the caregiving literature.**

|  | Self-Reported Difficulty Coordinating Care | | | |
| --- | --- | --- | --- | --- |
|  | No Difficulty  Weighted N: 1,567,324  Weighted % | Any Difficulty  Weighted N: 1,182,742  Weighted % | Total  Weighted N: 2,750,066  Weighted % | P-value |
| Overall | 57.0% | 43.0% | 100.0% |  |
| Caregiver age |  |  |  |  |
| 17-59 | 38.4% | 44.1% | 40.9% | 0.74 |
| 60-69 | 33.4% | 32.2% | 32.9% |  |
| c70 | 28.1% | 23.7% | 26.2% |  |
| Caregiver sex |  |  |  |  |
| Male | 27.6% | 40.0% | 32.9% | 0.078 |
| Female | 72.4% | 60.0% | 67.1% |  |
| Caregiver race and ethnicity |  |  |  |  |
| Non-Hispanic White | 59.6% | 52.0% | 56.3% | 0.028 |
| Black | 19.3% | 9.0% | 14.9% |  |
| Hispanic and Others | 21.1% | 39.0% | 28.8% |  |
| Co-residence with care recipient |  |  |  |  |
| No | 25.4% | 34.9% | 29.5% | 0.27 |
| Yes | 74.6% | 65.1% | 70.5% |  |
| Relationship to care recipient |  |  |  |  |
| Kin caregiver | 79.4% | 80.1% | 79.7% | 0.94 |
| Non-kin caregiver | 20.6% | 19.9% | 20.3% |  |
| ≥4 years of caregiving |  |  |  |  |
| No | 26.1% | 17.6% | 22.4% | 0.36 |
| Yes | 73.9% | 82.4% | 77.6% |  |
| Caregiver education |  |  |  |  |
| ≤ High school | 39.9% | 32.1% | 36.6% | 0.34 |
| ≥ Some college | 60.1% | 67.9% | 63.4% |  |
| Care recipient under Medicare Part D |  |  |  |  |
| No | 29.3% | 44.3% | 35.6% | 0.17 |
| Yes | 70.7% | 55.7% | 64.4% |  |
| Care recipient under Medigap |  |  |  |  |
| No | 41.5% | 44.5% | 42.8% | 0.74 |
| Yes | 58.5% | 55.5% | 57.2% |  |
| Care recipient under Medicaid |  |  |  |  |
| No | 65.0% | 61.6% | 63.5% | 0.71 |
| Yes | 35.0% | 38.4% | 36.5% |  |
| Telehealth use with regular doctor |  |  |  |  |
| No regular doctor | 4.8% | 6.8% | 5.7% | 0.73 |
| In person only | 83.4% | 80.1% | 82.0% |  |
| Online only or hybrid | 11.8% | 13.1% | 12.3% |  |
| Caregiving hours last month (mean, SE)* | 250.7 (267.2) | 178.5 (205.4) | 219.6 (245.0) | 0.059 |
| Care recipient’s number of chronic conditions (mean, SE)† | 3.0 (1.6) | 3.3 (1.7) | 3.1 (1.7) | 0.36 |
| Caregiver’s number of chronic conditions (mean, SE)‡ | 1.9 (1.6) | 1.8 (1.5) | 1.9 (1.6) | 0.61 |

*SE: standard error.

†Chronic conditions were self-reported, including heart attack, heart disease, high blood pressure, arthritis, osteoporosis, diabetes, lung disease, stroke, and cancer. SE: standard error.

‡Chronic conditions were self-reported, including heart attack, heart disease, high blood pressure, arthritis, osteoporosis, diabetes, lung disease, cancer, serious difficulty seeing, and serious difficulty hearing. SE: standard error.

**Supplementary Table S2a. Weighted Characteristics of Dementia Family Caregivers by Self-Reported Difficulty Coordinating Care (NSOC Round 13).**

|  | Self-Reported Difficulty Handling Health Insurance | | | |
| --- | --- | --- | --- | --- |
|  | No Difficulty  Weighted N: 842,224  Weighted % | Any Difficulty  Weighted N: 556,374  Weighted % | Total  Weighted N: 1,398,598  Weighted % | P-value |
| Overall | 60.2% | 39.8% | 100.0% |  |
| Caregiver age |  |  |  |  |
| 17-59 | 36.0% | 59.8% | 45.5% | 0.040 |
| 60-69 | 36.6% | 21.9% | 30.7% |  |
| ≥70 | 27.4% | 18.3% | 23.8% |  |
| Caregiver sex |  |  |  |  |
| Male | 30.1% | 54.3% | 39.7% | 0.051 |
| Female | 69.9% | 45.7% | 60.3% |  |
| Caregiver race and ethnicity |  |  |  |  |
| Non-Hispanic White | 61.5% | 63.2% | 62.1% | 0.50 |
| Black | 12.7% | 7.4% | 10.6% |  |
| Hispanic and Others | 25.9% | 29.4% | 27.3% |  |
| Co-residence with care recipient |  |  |  |  |
| No | 25.1% | 24.9% | 25.0% | 0.99 |
| Yes | 74.9% | 75.1% | 75.0% |  |
| Relationship to care recipient |  |  |  |  |
| Kin caregiver | 90.3% | 81.1% | 86.7% | 0.11 |
| Non-kin caregiver | 9.7% | 18.9% | 13.3% |  |
| ≥4 years of caregiving |  |  |  |  |
| No | 22.4% | 19.8% | 21.4% | 0.82 |
| Yes | 77.6% | 80.2% | 78.6% |  |
| Caregiver education |  |  |  |  |
| ≤ High school | 29.2% | 49.9% | 37.4% | 0.054 |
| ≥ Some college | 70.8% | 50.1% | 62.6% |  |
| Care recipient under Medicare Part D |  |  |  |  |
| No | 31.6% | 28.3% | 30.2% | 0.73 |
| Yes | 68.4% | 71.7% | 69.8% |  |
| Care recipient under Medigap |  |  |  |  |
| No | 27.8% | 32.2% | 29.7% | 0.70 |
| Yes | 72.2% | 67.8% | 70.3% |  |
| Care recipient under Medicaid |  |  |  |  |
| No | 62.9% | 53.3% | 59.1% | 0.42 |
| Yes | 37.1% | 46.7% | 40.9% |  |
| Telehealth use with regular doctor |  |  |  |  |
| No regular doctor | --* | 3.8% | 2.7% | 0.32 |
| In person only | 85.8% | 76.9% | 82.2% |  |
| Online only or hybrid | --* | 19.3% | 15.0% |  |
| Caregiving hours last month (mean, SE)† | 244.2 (247.3) | 273.2 (278.5) | 255.8 (259.8) | 0.64 |
| Care recipient’s number of chronic conditions (mean, SE)‡ | 2.9 (1.6) | 4.0 (2.0) | 3.3 (1.9) | 0.059 |
| Caregiver’s number of chronic conditions (mean, SE)§ | 2.0 (1.6) | 2.1 (1.7) | 2.0 (1.6) | 0.67 |

* Estimates based on too few cases (<5 unweighted cell count) may not be reported, per NHATS.

†SE: standard error.

‡Chronic conditions were self-reported, including heart attack, heart disease, high blood pressure, arthritis, osteoporosis, diabetes, lung disease, stroke, and cancer. SE: standard error.

§Chronic conditions were self-reported, including heart attack, heart disease, high blood pressure, arthritis, osteoporosis, diabetes, lung disease, cancer, serious difficulty seeing, and serious difficulty hearing. SE: standard error.

**Supplementary Table S2b. Weighted Characteristics of Dementia Family Caregivers by Self-Reported Difficulty Handling Health Insurance (NSOC Round 13).**

|  | Self-Reported Difficulty Transition after Overnight Hospital Stay | | | |
| --- | --- | --- | --- | --- |
|  | No Difficulty  Weighted N: 552,119  Weighted % | Any Difficulty  Weighted N: 792,053  Weighted % | Total  Weighted N: 1,344,172  Weighted % | P-value |
| Overall | 41.1% | 58.9% | 100.0% |  |
| Caregiver age |  |  |  |  |
| 17-59 | 35.1% | 46.1% | 41.6% | 0.44 |
| 60-69 | 35.2% | 34.0% | 34.5% |  |
| ≥70 | 29.7% | 19.9% | 23.9% |  |
| Caregiver sex |  |  |  |  |
| Male | 42.6% | 40.6% | 41.4% | 0.88 |
| Female | 57.4% | 59.4% | 58.6% |  |
| Caregiver race and ethnicity |  |  |  |  |
| Non-Hispanic White | 46.0% | 49.5% | 48.0% | 0.89 |
| Black | 15.1% | 14.9% | 15.0% |  |
| Hispanic and Others | 38.9% | 35.6% | 36.9% |  |
| Co-residence with care recipient |  |  |  |  |
| No | 28.0% | 24.4% | 25.9% | 0.73 |
| Yes | 72.0% | 75.6% | 74.1% |  |
| Relationship to care recipient |  |  |  |  |
| Kin caregiver | 89.3% | 81.1% | 84.4% | 0.27 |
| Non-kin caregiver | 10.7% | 18.9% | 15.6% |  |
| ≥4 years of caregiving |  |  |  |  |
| No | 27.1% | 27.4% | 27.3% | 0.98 |
| Yes | 72.9% | 72.6% | 72.7% |  |
| Received training post hospital stay |  |  |  |  |
| No | 37.2% | 45.0% | 41.8% | 0.41 |
| Yes | 62.8% | 55.0% | 58.2% |  |
| Caregiver education |  |  |  |  |
| ≤ High school | 34.0% | 44.6% | 40.3% | 0.40 |
| ≥ Some college | 66.0% | 55.4% | 59.7% |  |
| Care recipient under Medicare Part D |  |  |  |  |
| No | 32.5% | 35.9% | 34.6% | 0.80 |
| Yes | 67.5% | 64.1% | 65.4% |  |
| Care recipient under Medigap |  |  |  |  |
| No | 29.3% | 41.1% | 36.6% | 0.19 |
| Yes | 70.7% | 58.9% | 63.4% |  |
| Care recipient under Medicaid |  |  |  |  |
| No | 59.6% | 69.7% | 65.7% | 0.33 |
| Yes | 40.4% | 30.3% | 34.3% |  |
| Telehealth use with regular doctor |  |  |  |  |
| No regular doctor | 8.3% | 3.6% | 5.5% | 0.41 |
| In person only | 81.7% | 87.4% | 85.0% |  |
| Online only or hybrid | 10.0% | 9.0% | 9.4% |  |
| Caregiving hours last month (mean, SE)* | 237.1 (271.0) | 288.0 (255.3) | 267.1 (266.2) | 0.40 |
| Care recipient’s number of chronic conditions (mean, SE)† | 3.4 (2.1) | 3.5 (1.6) | 3.4 (1.8) | 0.86 |
| Caregiver’s number of chronic conditions (mean, SE)‡ | 2.0 (2.0) | 2.1 (1.3) | 2.1 (1.6) | 0.90 |

*SE: standard error.

†Chronic conditions were self-reported, including heart attack, heart disease, high blood pressure, arthritis, osteoporosis, diabetes, lung disease, stroke, and cancer. SE: standard error.

‡Chronic conditions were self-reported, including heart attack, heart disease, high blood pressure, arthritis, osteoporosis, diabetes, lung disease, cancer, serious difficulty seeing, and serious difficulty hearing. SE: standard error.

**Supplementary Table S2c. Weighted Characteristics of Dementia Family Caregivers by Self-Reported Difficulty Transitioning after Overnight Hospital Stay (NSOC Round 13).**
